# Volumetric modulated arc therapy treatment planning based on virtual monochromatic images for head and neck cancer: effect of the contrast‐enhanced agent on dose distribution

**DOI:** 10.1002/acm2.12752

**Published:** 2019-10-21

**Authors:** Riho Komiyama, Shingo Ohira, Naoyuki Kanayama, Tsukasa Karino, Hayate Washio, Yoshihiro Ueda, Masayoshi Miyazaki, Teruki Teshima

**Affiliations:** ^1^ Department of Radiation Oncology Osaka International Cancer Institute Osaka Japan; ^2^ Department of Medical Physics and Engineering Osaka University Graduate School of Medicine Suita Japan

**Keywords:** contrast agent, dual energy CT, head and neck cancer, virtual monochromatic image

## Abstract

Virtual monochromatic images (VMIs) at a lower energy level can improve image quality but the computed tomography (CT) number of iodine contained in the contrast‐enhanced agent is dramatically increased. We assessed the effect of the use of contrast‐enhanced agent on the dose distributions in volumetric modulated arc therapy (VMAT) planning for head and neck cancer (HNC). Based on the VMIs at 40 keV (VMI_40keV_), 60 keV(VMI_60keV_), and 77 keV (VMI_77keV_) of a tissue characterization phantom, lookup tables (LUTs) were created. VMAT plans were generated for 15 HNC patients based on contrast‐enhanced‐ (CE‐) VMIs at 40‐, 60‐, and 77 keV using the corresponding LUTs, and the doses were recalculated based on the noncontrast‐enhanced‐ (nCE‐) VMIs. For all structures, the difference in CT numbers owing to the contrast‐enhanced agent was prominent as the energy level of the VMI decreased, and the mean differences in CT number between CE‐ and nCE‐VMI was the largest for the clinical target volume (CTV) (125.3, 55.9, and 33.1 HU for VMI_40keV_, VMI_60keV_, and VMI_77keV,_ respectively). The mean difference of the dosimetric parameters (D_99%_, D_50%_, D_1%_, D_mean_, and D_0.1cc_) for CTV and OARs was <1% in the treatment plans based on all VMIs. The maximum difference was observed for CTV in VMI_40keV_ (2.4%), VMI_60keV_ (1.9%), and VMI_77keV_ (1.5%) plans. The effect of the contrast‐enhanced agent was larger in the VMAT plans based on the VMI at a lower energy level for HNC patients. This effect is not desirable in a treatment planning procedure.

## 1 INTRODUCTION

For several years, volumetric modulated arc therapy (VMAT), with continuously varying gantry rotation speed, multileaf collimator (MLC) pattern, and dose rate during delivery, has been introduced in clinical radiation oncology to control tumor growth by delivering a high dose to the tumor and/or reduce the risk of normal tissue injury.^1^ Head‐and‐neck cancer (HNC) patients are expected to benefit from the VMAT technique because the tumors are often irregularly shaped and are surrounded by many organs at risk (OARs).^2^, ^3^, ^4^ For such sophisticated treatment, computed tomography (CT) simulation plays an important role for accurate target delineation and dose calculation. Commonly, CT numbers (Hounsfield unit, HU) are converted into electron density during dose calculation to account for the inhomogeneous environment in a patient anatomy.

In modern radiotherapy, treatment plans are commonly generated on CT, and an intravenous contrast‐enhanced agent is used for delineating the tumor and OARs for HNC patients. Recently dual‐energy CT (DECT), which utilizes two different energy spectra, was used for the reconstruction of virtual monochromatic images (VMIs) at a given photon‐energy level (usually 40–140 keV).^5^, ^6^ Wichmann et al. reported that the VMIs at 60 keV significantly improve the contrast noise ratio (CNR), subjective overall image quality, and tumor delineation of head and neck squamous cell carcinoma.^7^ According to another report by Lam *et al.*, the difference in CT number between tumor and muscle is the highest on the VMI at 40 keV for HNC.^8^ The high quality of image has the potential for improving the accuracy of target/OARs delineation for radiotherapy treatment planning.^9^, ^10^

The CT numbers in contrast‐enhanced VMI (CE‐ VMI) are higher than those in noncontrast‐enhanced VMI (nCE‐ VMI) when patients are treated without using the contrast‐enhanced agent. Especially, the CT number of iodine contained in the contrast‐enhanced agent is markedly increased in the VMI at a low energy level due to the K‐shell photon absorption.^11^ Although the VMI at a low energy level provides a high image quality, the difference in CT number between CE‐ and nCE‐VMI was prominent as the energy level of the VMI decreased.^8^ These physical characteristics of VMI can affect the treatment planning in the dose calculation process, and unexpected radiation side effect can occur. Alan et al. evaluated the effect of contrast‐enhanced agent in the dose distribution of the VMAT for the HNC using 120 kVp images.^12^ However, to the best of our knowledge, no previous studies have investigated the effect of contrast‐enhanced agent based on the difference of energy levels in the VMIs.

The purpose of this study was to assess the effect of the use of contrast‐enhanced agent on the VMAT dose distributions based on VMIs at different energy level for HNC. This study compares the dosimetric parameters (D_99%_, D_50%_, D_1%_, D_mean_, and D_0.1cc_) between the treatment plans generated using CE‐ and nCE‐VMI at three energy levels: 40 (high‐contrast), 60 (high image quality), and 77 (equivalent CT numbers with 120 kVp images 13 keV).

## 2 MATERIALS AND METHODS

### 2.1 Electron density conversion table

**Figure 1:**
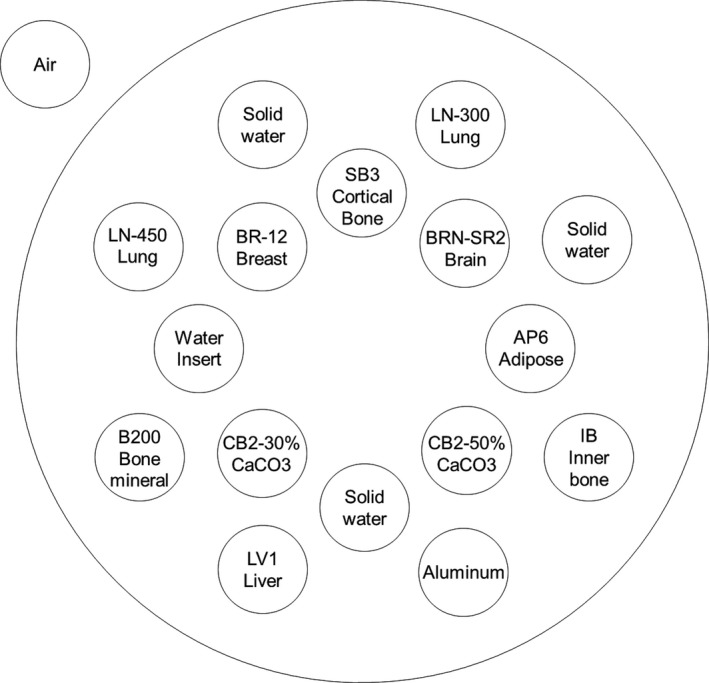
Arrangement of the reference materials of the tissue characterization phantom.

The electron density relative to water (ED) lookup table (LUT) was generated using a tissue characterization phantom (GAMMEX467, Gammex RMI, Middleton, WI), which is used in conjunction with a CT scanner to establish the relationship between the ED of various tissues and their corresponding CT numbers in HU. The reference materials mimicked human body organs with known EDs, and the specifications are listed in Table 1. To extend the usable range of the LUT, reference material made of aluminum was used in this study. The arrangement of the reference materials of the tissue characterization phantom are shown in Fig. 1. The DECT scans (Revolution HD, GE Healthcare, Milwaukee, WI) were performed using 80/140 kVp photon beam energies, and the measurement was repeated five times to minimize random variations of the HU measurement. Based on the acquired data, VMIs at 40, 60, and 77 keV (VMI_40keV,_ VMI_60keV_, and VMI_77keV,_ respectively) were reconstructed, and the scanning parameters were as follows: helical pitch: 0.984:1, field of view (FOV): 500 mm, slice thickness: 2 mm, and volume CT dose index: 15.02 mGy. The theoretical ED was plotted as a function of the mean CT numbers in VMI_40keV,_ VMI_60keV_, and VMI_77keV_, and the LUT for each VMI was registered in a treatment planning system (TPS) (Eclipse version 13.7, Varian Medical Systems, Palo Alto, CA).

**Table 1:** Specifications of the reference materials of the phantom.

	Electron density	CT number		
		40 keV	60 keV	77 keV
Air	0.001	−993	−997.6	−998.8
Lung 300	0.278	−631.2	−672.6	−689
Lung 450	0.455	−484.9	−512	−523.6
Adipose	0.932	−148.5	−94.2	−76.7
Breast	0.96	−19.5	−27.9	−31.1
SW1	0.987	31.3	8.7	3.8
SW2	0.987	18.2	4.8	0.8
SW3	0.987	32.8	10.9	4.4
Water	1	35.8	11.9	3.5
Brain	1.047	42.9	39.5	41.3
Liver	1.06	63.6	63.5	66.1
Inner bone	1.096	557.3	295.2	205.7
B200	1.104	561.6	301.7	213
CB30%	1.277	854.5	526.2	413.4
CB50%	1.469	1663.8	988.1	754.3
SB3	1.694	2630.5	1531.8	1151.2
Aluminum	2.36	3018.2	2155.4	1795.6

### 2.2 CT scans

This retrospective study included 15 patients (median (range) age 62 (50–76) yr; 11 male and 4 female) treated using VMAT technique at our institution. The primary site was oropharynx in six patients, hypopharynx in three, larynx in three, nasopharynx in one, maxillary sinus in one, and oral cavity in one. The study was approved by our ethics committee with written informed consent provided by the patients. The patients were immobilized with a thermo plastic mask in a supine position. Two consecutive DECT scans were performed for each patient. The first DECT set was scanned before the contrast‐enhanced agent was injected (nCE‐VMI). The second DECT acquisition was performed with the injection of the contrast‐enhanced agent to achieve 450 mgI/kg with an injection time of 50 s (1.5 to 1.9 ml/s), and the patients were scanned for 70 s after the injection (CE‐VMI). Based on the acquired data, CE‐ and nCE‐ VMI_40keV,_ and VMI_60keV,_ and VMI_77keV_ were reconstructed with a slice thickness of 2 mm, FOV of 500 mm, and a matrix size of 512 × 512 and were acquired with a helical pitch of 0.984:1 and volume CT dose index of 15.02 mGy.

### 2.3 Objective image analysis

The CT image series of the HNC patient were transferred to a workstation (Advantage Sim, GE Healthcare, Milwaukee, WI) for objective image analysis. For the tumor and sternocleidomastoid muscle, three nonoverlapping circular regions of interest (ROIs) (diameter of 5 mm) were placed, and the CT numbers and standard deviation (SD) were measured within the ROIs in each patient. The focal areas of tumor necrosis were avoided to include only the enhanced lesion areas. The CNR was calculated as (mean CT number of tumor – mean CT number of muscle)/((SD of tumor)^2^ + (SD of muscle)^2^)^1/2^.

### 2.4 Treatment plan

CE‐VMIs were then transferred to a TPS, and target volumes and OARs (i.e., oral cavity, parotid ipsilateral, parotid contralateral, brainstem, and spinal cord) were contoured by radiation oncologists. In accordance with the recommendations in Reports 50 and 62 of the International Commission on Radiation Units, the gross tumor volume (GTV), clinical target volume (CTV), and planning target volume (PTV) were determined. The elective CTV (CTVe) and boost CTV (CTVb) were delineated, with 0.3–0.5 cm margins applied for the elective PTV (PTVe) and boost PTV (PTVb), respectively.^14^

**Figure 2:**
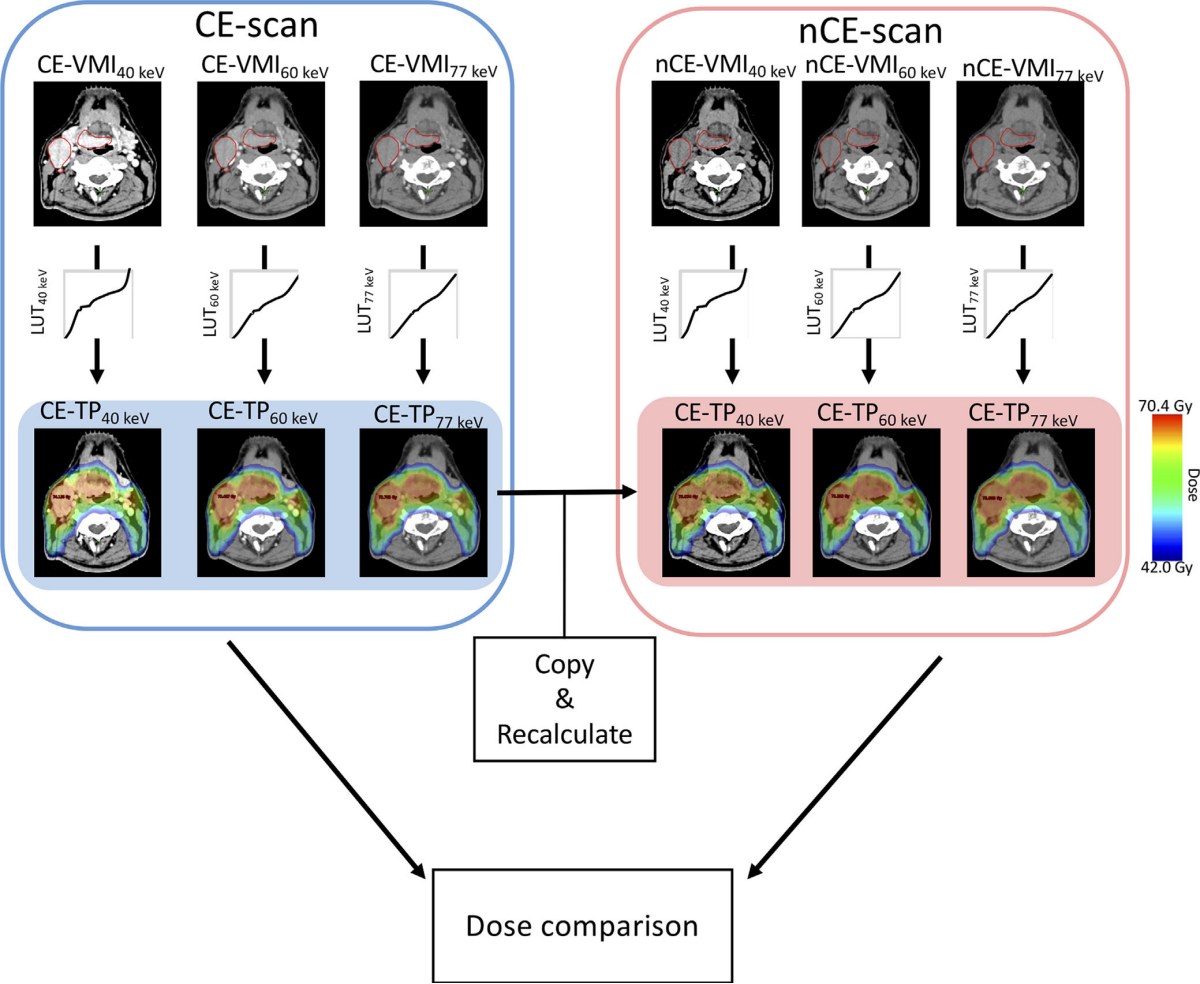
Schematic workflow of the radiotherapy treatment planning based on the virtual monochromatic image and dose comparison.

Figure 2 illustrates a schematic work flow in this study for radiotherapy treatment planning based on the DECT images. VMAT treatment planning was performed based on CE‐ VMI_40keV,_ VMI_60keV_, and VMI_77keV_ using the corresponding LUTs (LUT_40keV_, LUT_60keV_, and LUT_77keV_) to deliver 70.4 and 46.0 Gy for PTVb and PTVe, respectively. The following dose constraints were used for OARs: mean dose (D_mean_) for oral cavity: <40 Gy; D_mean_ for parotid (at least one): <26 Gy; maximal dose (D_max_) for brainstem: <54 Gy; D_max_ for spinal cord: <45 Gy.

All treatment plans were generated using a photon beam energy of 6 MV and the doses were calculated using the Anisotropic Analytical Algorithm (AAA) and Acuros XB (AXB) with a dose grid size of 2 mm. To evaluate the dose difference between the treatment plans based on CE‐ and nCE‐VMI, each treatment plan based on CE‐ VMI was copied into the corresponding nCE‐ VMI with all planning parameters remaining constant (MU, MLC movement, etc.), and the dose was recalculated. The structures of the targets and OARs on the CE‐ VMI were transferred to the nCE‐ VMI after bony registration. The dosimetric parameter difference for the targets and OARs was determined as the percent difference in dosimetric parameters in the CE‐TP (CE‐ treatment plan) relative to the corresponding values in the nCE‐TP (nCE‐ treatment plan). Wilcoxon paired signed rank test was performed to measure any significant difference in the dosimetric parameters of the targets and OARs between treatment plans based on CE‐ VMI and nCE‐ VMI (SPSS, version 24; IBM Corp., Armonk, NY). A *P *< 0.05 was considered statistically significant.

## 3 RESULT

**Figure 3:**
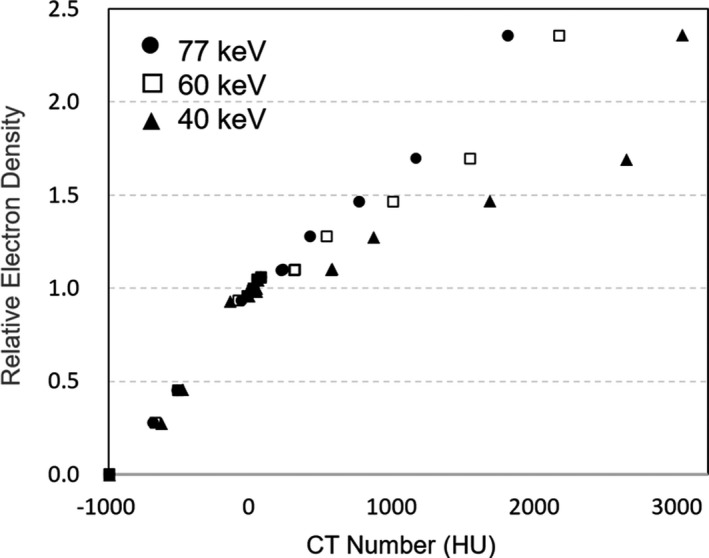
Lookup tables generated from the VMI_40keV,_ VMI_60keV_, and VMI_77keV_.

Figure 3 shows the respective LUTs generated from the VMI_40keV_, VMI_60keV_, and VMI_77keV_(LUT_40keV_, LUT_60keV_, and LUT_77keV_). The equivalent CT numbers were obtained for the low‐density materials in LUT_40keV_, LUT_60keV_, and LUT_77keV_ (ED < 0). The CT number changed considerably for high‐density material rods, and the CT number of aluminum varied widely in the range from 1796 ± 55.9 HU (VMI_77keV_) to 3018 ± 49.3 HU (VMI_40keV_).

**Figure 4:**
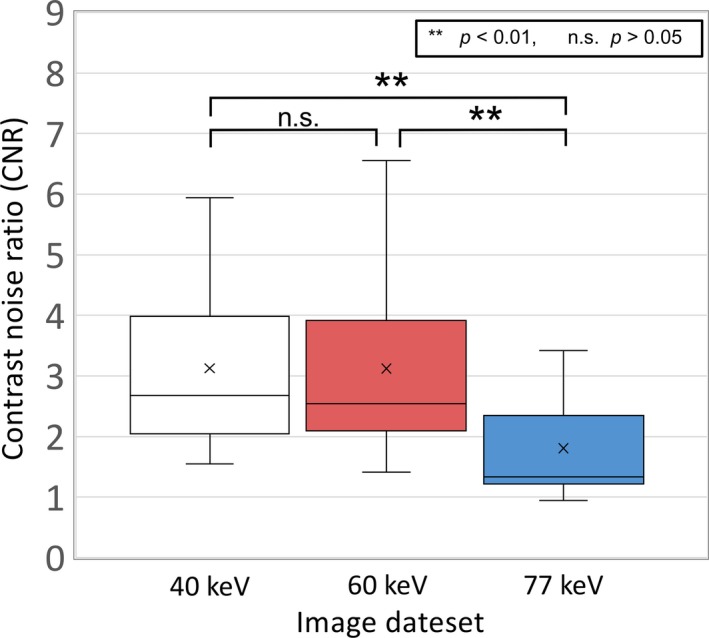
The contrast noise ratios (CNRs) on the VMI_40keV,_ VMI_60keV_, and VMI_77keV_.

The box and whiskers plot of the CNR in the VMIs at 40, 60, and 77 keV are shown in Fig. 4. The mean values (±SD) of the CNR at 40, 60, and 77 keV were 3.1 ± 1.5, 3.1 ± 1.6, and 1.8 ± 0.8, respectively. The CNR at VMI_77 keV_ was significantly lower than those of the other energy levels (40 and 60 keV). Table 2 shows the mean and standard deviation (SD) of the CT numbers in the VMI with and without the contrast‐enhanced agent (CE‐ and nCE‐VMI) for the targets (CTVb and CTVe) and OARs. For all structures, the CE‐VMI resulted in significantly higher CT numbers than those in the nCE‐VMI (*P* < 0.01), and the difference in the CT numbers owing to the contrast‐enhanced agent was prominent as the energy level of the VMI decreased. For the CTVb, the CT numbers of the CE‐VMI_40keV_ and nCE‐VMI_40keV_ were 206.1 ± 98.8 and 80.8 ± 109.3 HU, and those of the CE‐VMI_60keV_ and nCE‐VMI_60keV_ were 109.9 ± 73.2 and 54.0 ± 78.5 HU, while those of the CE‐VMI_77keV_ and nCE‐VMI_77keV_ were 77.6 ± 62.2 and 44.5 ± 66.1 HU, respectively. In contrast, the CE and nCE‐VMIs provided the equivalent CT numbers for the brainstem (CE‐VMI_40keV_ and nCE‐VMI_40keV_: 71.6 ± 22.1 and 56.8 ± 9.5 HU; CE‐VMI_60keV_ and nCE‐VMI_60keV_: 44.7 ± 4.2 and 37.0 ± 3.6; CE‐VMI_77keV_ and nCE‐VMI_77keV_: 34.2 ± 2.7 and 30.2 ± 2.0 HU). The ED and mass densities (MD) in the CE‐ and nCE‐TP for the target and OARs are shown in Table 3. Consequently, the CE‐VMI was observed to have higher or equivalent ED and MD than those in the nCE‐VMI (*P* < 0.01) for the targets and OARs (except for brain stem), as the energy level of the VMI decreased. The maximum difference of the ED between the CE‐VMI and nCE‐VMI was 0.8 for the CTVe(VMI_40keV_), and that of MD was 0.9 g/cm^3^ for the CTVe and parotid ipsilateral (VMI_40keV_).

**Table 2:** Computed tomography (CT) number of the contrast‐enhanced‐ (CE) and non‐CE (nCE)‐virtual monochromatic image (VMI) (40, 60, and 77 keV) in targets and organs at risks (OARs).

	40 keV		60 keV		77 keV	
	Average	SD	Average	SD	Average	SD
CTVboost						
CE	206.1	98.3	109.9	73.2	77.6	62
nCE	80.8	109.3	54	78.5	44.5	66.1
*P*	0.001		0.001		0.001	
CTVelective						
CE	120.8	58.6	56.2	34.3	32.3	25.9
nCE	4.9	64.9	4.5	38.6	3.3	29.3
*P*	0.001		0.001		0.001	
Oral cavity						
CE	164.5	110	79.4	75.2	43	66.3
nCE	103.8	107.5	45.3	80.8	23.3	72
*P*	0.001		0.001		0.003	
Parotid contralateral						
CE	70.2	62.6	35.9	35.8	24	27.5
nCE	−39.4	50.4	−14	29.6	−3.5	24.2
*P*	0.001		0.001		0.001	
Parotid ipsilateral						
CE	76.3	47.9	38.3	27.6	36.4	47.9
nCE	−39.8	38.6	−12.4	26.3	−4.6	22.1
*P*	0.001		0.001		0.001	
Brain stem						
CE	71.6	22.1	44.7	4.2	34.2	2.7
nCE	56.8	9.5	37	3.6	30.2	2
*P*	0.001		0.001		0.001	
Spinal cord						
CE	76.8	27.3	54.1	16.2	41.1	12.3
nCE	49.8	27.1	45.9	14.2	37.4	11.2
*P*	0.001		0.001		0.001	

**Table 3:** Electron density relative to water (ED) and Mass density (MD) of the contrast‐enhanced‐ (CE) and non‐CE (nCE)‐virtual monochromatic image (VMI) (40, 60, and 77 keV) in targets and organs at risks (OARs).

	40 keV		60 keV		77 keV	
	ED	MD (g/cm^3^)	ED	MD (g/cm^3^)	ED	MD (g/cm^3^)
CTVboost						
CE	1.07 ± 0.01	1.11 ± 0.01	1.07 ± 0.02	1.10 ± 0.03	1.06 ± 0.03	1.09 ± 0.04
nCE	1.04 ± 0.04	1.06 ± 0.04	1.04 ± 0.04	1.06 ± 0.04	1.03 ± 0.04	1.05 ± 0.05
*P*	0.001	0.001	0.001	0.001	0.001	0.001
CTVelective						
CE	1.06 ± 0.00	1.10 ± 0.01	1.05 ± 0.02	1.07 ± 0.03	1.02 ± 0.02	1.04 ± 0.03
nCE	0.98 ± 0.04	1.01 ± 0.04	0.99 ± 0.03	1.01 ± 0.03	0.99 ± 0.03	1.02 ± 0.03
*P*	0.001	0.001	0.001	0.001	0.001	0.001
Oral cavity						
CE	1.06 ± 0.03	1.10 ± 0.03	1.04 ± 0.04	1.07 ± 0.05	1.03 ± 0.05	1.05 ± 0.06
nCE	1.04 ± 0.05	1.07 ± 0.05	1.02 ± 0.05	1.05 ± 0.06	1.01 ± 0.05	1.03 ± 0.06
*P*	0.001	0.001	0.001	0.001	0.001	0.002
Parotid contralateral						
CE	1.03 ± 0.04	1.06 ± 0.05	1.03 ± 0.04	1.05 ± 0.04	1.02 ± 0.03	1.04 ± 0.03
nCE	0.96 ± 0.03	0.99 ± 0.03	0.98 ± 0.03	1.00 ± 0.03	0.99 ± 0.03	1.01 ± 0.02
*P*	0.001	0.001	0.001	0.001	0.001	0.001
Parotid ipsilateral						
CE	1.05 ± 0.03	1.07 ± 0.04	1.03 ± 0.03	1.05 ± 0.03	1.02 ± 0.03	1.05 ± 0.04
nCE	0.96 ± 0.03	0.98 ± 0.03	0.98 ± 0.03	1.00 ± 0.02	0.99 ± 0.02	1.01 ± 0.02
*P*	0.001	0.001	0.001	0.001	0.001	0.006
Brain stem						
CE	1.05 ± 0.02	1.09 ± 0.02	1.05 ± 0.00	1.06 ± 0.01	1.04 ± 0.00	1.04 ± 0.00
nCE	1.05 ± 0.01	1.07 ± 0.02	1.04 ± 0.01	1.05 ± 0.00	1.03 ± 0.00	1.04 ± 0.00
*P*	0.011	0.009	0.001	0.001	0.001	0.001
Spinal cord						
CE	1.06 ± 0.01	1.09 ± 0.02	1.05 ± 0.01	1.07 ± 0.01	1.04 ± 0.01	1.05 ± 0.02
nCE	1.04 ± 0.03	1.06 ± 0.03	1.05 ± 0.01	1.06 ± 0.02	1.04 ± 0.01	1.05 ± 0.01
*P*	0.001	0.001	0.001	0.001	0.001	0.001

**Figure 5:**
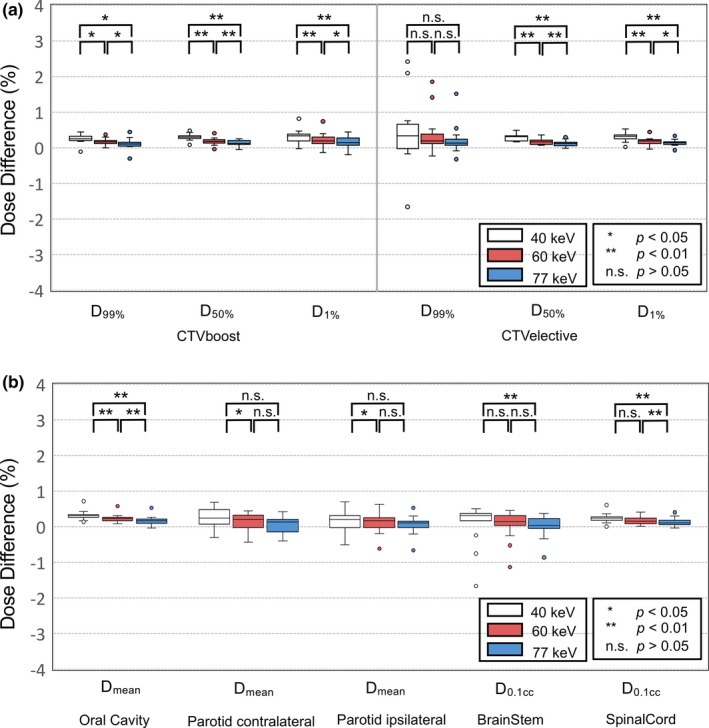
Difference in the dosimetric parameters between the treatment plans using Anisotropic Analytical Algorithm based on the contrast‐enhanced‐ (CE) and non‐CE (nCE)‐virtual monochromatic image for the (a) planning target volume and (b) organs at risks.

Figure 5 summarizes the relative differences in the dosimetric parameters using the AAA between the CE‐ and nCE‐TP for the target and OARs. Regarding the target, the CE‐TP had higher values of the dosimetric parameters than the nCE‐TP for almost all patients, and the mean difference in the dosimetric parameters was <1%. As the energy level of the VMI decreased, statistically significant differences in the dosimetric parameters (*P* < 0.05) were observed (except for D_99%_ in the CTVe). The maximum differences in D_1%_ for the CTVb were 0.8% and 0.7% in the VMI_40keV_ and VMI_60keV,_ respectively. For the CTVe, the maximum differences in the VMI_40keV_, VMI_60keV_, and VMI_77keV_ were 2.4%, 1.9%, and 1.5% in D_99%_, respectively. For the OAR, a similar trend of differences in the dosimetric parameters was observed as that for the targets. The maximum difference was −1.7% for the brainstem (D_0.1cc_) in the VMI_40keV_, −1.1% in the VMI_60keV_, and −0.9% in the VMI_77keV_.

**Figure 6:**
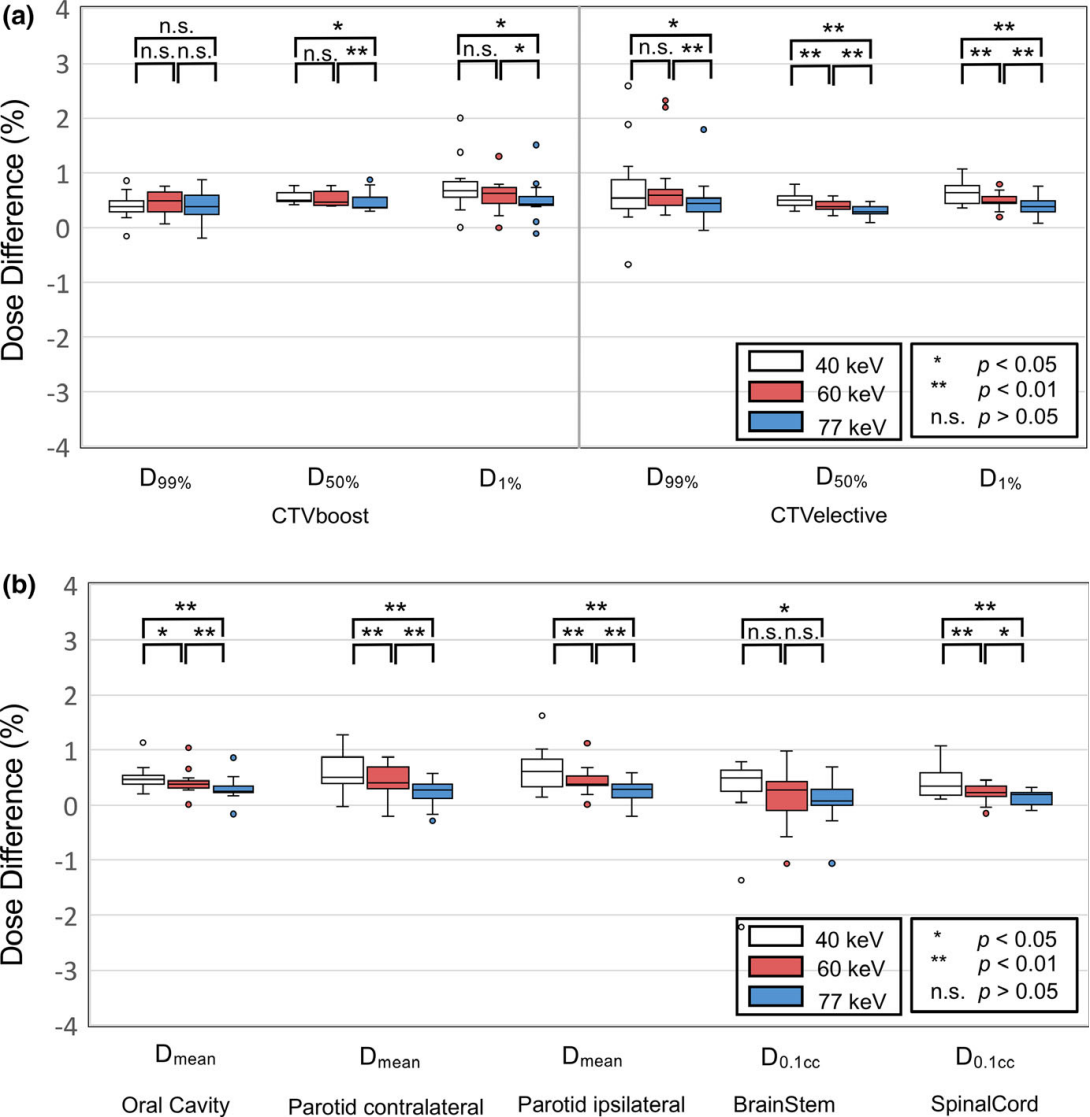
Difference in the dosimetric parameters between the treatment plans using Acuros XB based on the contrast‐enhanced‐ (CE) and non‐CE (nCE)‐virtual monochromatic image for the (a) planning target volume and (b) organs at risks.

The relative differences between the CE‐ and nCE‐TP for the target and OARs in the dosimetric parameters using the AXB are shown in Fig. 6. For the target and OAR, the mean difference in the dosimetric parameters was <1%. A similar trend of difference in the dosimetric parameters was observed as those of the AAA. The maximum differences in D_1%_ were 2.0%, 1.3%, and 1.5% in VMI_40keV_, VMI_60keV,_ and VMI_77keV,_ respectively. The maximum differences for the CTVe in VMI_40keV_, VMI_60keV_, and VMI_77keV_ were 2.6%, 2.3%, and 1.7% in D_99%_, respectively. For the OAR, the maximum difference was −2.2% for the brainstem (D_0.1cc_) in VMI_40keV_, −1.1% in VMI_60keV_, and −1.1% in VMI_77keV_.

## 4 DISCUSSION

In this study, we demonstrated that the form of LUTs changed depending on the energy of VMI, and the CT number changed dramatically in the VMI at low‐energy levels, particularly for a high‐density material. At low‐energy levels, the photoelectric effect is the dominant interaction, which is probability proportional to the atomic number cubed, and the Compton effect becomes the dominant interaction as the photon energy increase.^13^ Thus, particularly, the CT number of the high‐density material with a high atomic number varied widely depending on the energy level of the VMI. It should be remembered that these characteristics of the VMI and the appropriate LUTs should be assigned to the corresponding VMI for treatment planning.^15^

In this study, for patients with the HNC, the differences in the CT number between the CE‐VMI and nCE‐VMI were the largest for the targets while the CT number varied slightly in the brainstem and spinal cord. The reason was explained by the fact that a hypervascular tumor takes up iodine more than the OARs such as brainstem and spinal cord.^16^ Lam et al. demonstrated that the tumor attenuation of the primary tumor for the HNC was highest in the VMI_40 keV_ (207.9 ± 46.8 HU).^8^ A similar CT number was observed in the VMI_40 keV_ for the CTVb in this study (Table 2). Further, the difference in the CT number between the CE‐ and nCE‐VMI were larger as the energy level of the VMI decreased. This phenomenon is explained by the fact that the x‐ray output energy at low tube voltages is closer to the iodine K‐edge of 33 keV.^17^, ^18^ Thus, the sudden increase of the CT number is found in the VMI at low‐energy levels. Furthermore, Lam et al. evaluated that the tumor‐muscle CNR was the highest at VMI_40keV_, and they concluded that tumor conspicuity is greatest at VMI_40 keV_ and is useful for tumor detection.^8^ In our study, the energy level of the VMI for highest CNR was the same as their study. Although the VMI at low‐energy levels with a high image quality may improve the accuracy of target delineation in radiotherapy treatment planning, the iodine does not exist in the patients during dose delivery.

Alan et al. evaluated the effect of contrast in the dosimetry of the VMAT for the HNC using a conventional 120 kVp image.^12^ In their study, treatment planning on contrasted images generally showed a lower dose administered to both organs and target than planning on noncontrasted images, and the difference is generally <2%. To the best of our knowledge, this is the first study to compare the VMAT plans generated using the VMIs between with and without the contrast‐enhanced agent on the dose calculations. The effect was significant as the energy level of the VMI decreased (Fig. 5) because the difference in the CT number between the CE‐ and nCE‐VMI was large in the VMI at a low‐energy level. In this study, the mean effect on the targets and OARs in the VMI_40keV_ was <1%, and the effect did not differ more than 2.5% in any patient. The main advantage of the AXB algorithm is its accuracy in practice of radiotherapy.^19^, ^20^ However, the impact of the contrast‐enhanced agent was larger in the AXB than AAA, as shown in Figs. 5, 6. We should be aware of the dose difference in the calculated target dose between CE‐VMI and nCE‐VMI when using the AXB algorithm. The effect of contrast‐enhanced agent on the accuracy of treatment planning has been studied in the past on conventional planning in various anatomical regions. Lees et al. observed that the increase of dose is <2% in the lung cancer treatment plans,^21^ and Li et al. demonstrated that the dose difference of the PTV was <1% in the treatment plans for patients with esophageal cancer.^22^ Therefore, the contrast‐enhanced agent potentially affects the dose calculations but the effect seems to be clinically insignificant. The difference of dose calculation due to the contrast‐enhanced agent should not appear. In recent years, a water density image (WDI), which illustrates the density of the object with suppressed iodine information and serves as a true‐unenhanced image,^23^ was reconstructed from the DECT system. Ohira et al. showed that the treatment planning based on the WDI reduced the effect of the contrast‐enhanced agent.^24^ The WDI may improve the accuracy of dose distributions in radiotherapy treatment planning by removing the iodine component from the contrast‐enhanced images for the VMAT plans for patients with the HNC.

There are several limitations of this study. First, the dose difference is not solely caused by the contrast agent. Although patients were immobilized by a thermoplastic mask, the dose difference could be caused by changing the patient position between the CE‐VMI and nCE‐VMI scans. There is literature on investigation of interfraction errors of a head‐and‐shoulder thermoplastic mask.^25^ Velec et al. reported that the evaluated intrafraction setup error measured by comparing the before‐IMRT delivery and postfraction CBCT scans, and the mean of the difference between before and after treatment was <1 mm in three degrees (medial–lateral, cranial–caudal, and anterior–posterior). In our study, the time taken between the nCE and CE scan was approximately 3 min, which is equal or shorter than that of treatment. Thus, the effect of the intrafraction error is considered to be small, and a medical physicist validated the registration and checked that there was no considerable anatomical change and rotation error between the CE and nCE scan. There also were no large streaking artifacts in the CE scans. Therefore, deformable image registration may be an effective tool for decreasing the dose difference by changing the patient position between the CE‐VMI and nCE‐VMI scans. Second, we used a single‐source DECT, which uses a single x‐ray tube with a fast kilovolt switching system and a single detector. However, there are several types of DECT acquisition systems: dual‐source DECT, which utilizes two x‐ray tubes and two detectors, and detector‐based spectral CT, which uses a single x‐ray tube and a detector made of two layers.^26^, ^27^ Thus, such difference in the DECT system may have a different impact on dose calculation. Third, we have not investigated the effect of beam hardening on the dose distribution. The CT number measured using the DECT scanner varied depending on the volume of the surrounding material according to the literature.^15^ For acquiring LUTs using the GAMMEX phantom, the effect of beam hardening could have different CT numbers depending on the ring used as the reference material made of aluminum, especially for the lower energy VMI. Our previous study demonstrated that the CT numbers in the case of VMIs at low energy levels could be considerably inaccurate, especially for high‐density materials.^15^ Because the CT numbers are converted into ED and MD values by using LUTs for dose calculation in the radiotherapy treatment planning processes, the inaccurate CT numbers could possibly affect the dose distributions. Finally, the effect of the contrast‐enhanced agent on the accuracy of the target and/or OAR contouring was not investigated in this study. The difference of contouring with and without the contrast‐enhanced agent, which has not been investigated in this study, may be the effect of dose distribution.

## 5 CONCLUSION

With the decrease of energy levels in the VMIs, the difference in the CT number between CE‐ and nCE‐VMI increased. The deviation of the CT number affected the dose calculation more significantly in the treatment plan using the VMI_40keV_ than that using the VMI_77keV_. Although the mean effect of the contrast‐enhanced agent was <1%, the maximum difference was 2.4% for evaluating the target dose in the treatment plan based on the VMI_40keV_. Such dosimetric differences should not occur in the treatment planning procedure.

## CONFLICT OF INTEREST

No conflict of interest.
